# The Impact of Vitamin D and Its Dietary Supplementation in Breast Cancer Prevention: An Integrative Review

**DOI:** 10.3390/nu16050573

**Published:** 2024-02-20

**Authors:** Antía Torres, Carla Cameselle, Paz Otero, Jesus Simal-Gandara

**Affiliations:** Nutrition and Bromatology Group, Department of Analytical Chemistry and Food Science, Faculty of Science, University de Vigo, E-32004 Ourense, Spain; antia.torres.garcia@uvigo.es (A.T.); carla.cameselle.llanos@uvigo.es (C.C.)

**Keywords:** vitamin D, breast cancer, prevention, optimal levels, mechanism of action, risk factors

## Abstract

Vitamin D deficiency is currently a significant public health issue closely linked to numerous diseases, such as breast cancer. This study aims to determine the estimated optimal serum levels of vitamin D to have a protective effect against breast cancer, in addition to exploring the biological mechanisms and risk factors involved. A literature search of articles published in the last 5 years was conducted, and simple statistical analyses using mean and standard deviation were performed to calculate the average concentration of vitamin D from different available studies. It has been observed that serum levels of vitamin D ≥ 40.26 ng/mL ± 14.19 ng/mL could exert a protective effect against breast cancer. Additionally, various biological mechanisms, such as those related to the immune system, and risk factors like diet implicated in this relationship were elucidated. Consequently, it can be concluded that proper serum levels of vitamin D may have a protective effect against breast cancer, and dietary supplementation may be an appropriate procedure to achieve these optimal vitamin D concentrations.

## 1. Introduction

**Figure 1:**
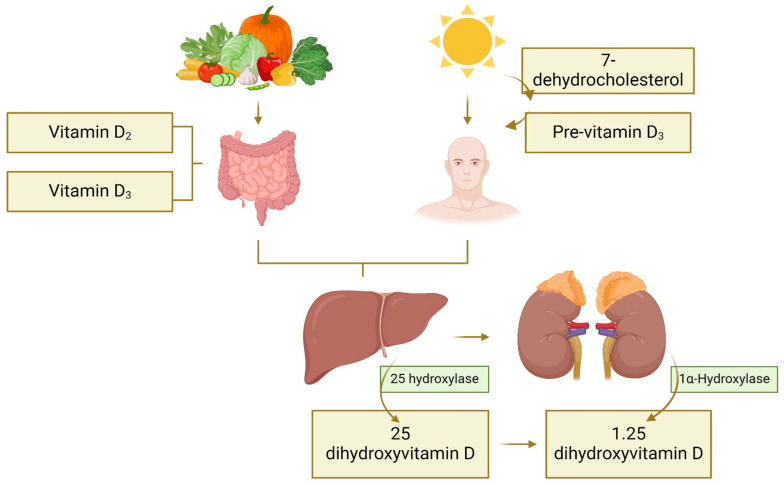
Scheme of the synthesis process of 1,25(OH)2D through ultraviolet-B (UVB) radiation and diet, divided into two stages: in the liver, vitamin D is converted to 25-hydroxyvitamin D by the action of 25-hydroxylases, and subsequently in the kidney, it is converted to 1,25-dihydroxyvitamin D by the action of 1-α hydroxylase.

Vitamin D, a group of fat-soluble vitamins renowned for their role in preserving the balance of calcium and phosphorus, is ubiquitous in virtually all tissues and cells of the human body [1]. Its extensive research has been fueled by its connection to various diseases, including different types of cancer [2]. The significance of the link between vitamin D and cancer, especially in the context of maintaining optimal serum levels for prevention, has grown substantially, given that an estimated 30% of adults grapple with a deficiency in vitamin D (serum 25-hydroxyvitamin D (25(OH)D) < 50 nmol/L), and over 60% exhibit insufficient levels (serum 25-hydroxyvitamin D (25(OH)D) 50–75 nmol/L) [3]. The root causes are likely multifaceted, encompassing socio-cultural practices that discourage sun exposure, dietary limitations, environmental pollution, a rise in obesity prevalence, and genetic factors [3]. A notable feature is its synthesis through exposure to ultraviolet-B (UVB) radiation from the sun [4], using the cholesterol precursor 7-dehydrocholesterol, which absorbs this radiation and transforms it into pre-vitamin D3. Subsequently, it undergoes thermal isomerization to become vitamin D3 [5]. Factors such as different skin types and the place of residence can influence this synthesis process [6]. Figure 1 represents this process. Additionally, vitamin D is obtained through dietary sources, although the available sources, such as fatty fish or certain fruits, are limited, and through supplements [7]. Regardless of its origin (vitamin D2 or vitamin D3), enzymatic hydroxylation in the liver produces 25-hydroxyvitamin D (25(OH)D), followed by further conversion in the kidney to 1,25-dihydroxyvitamin D2 or D3, known as calcitriol [8]. Calcitriol (1,25(OH)2D) is important for regulating the metabolism of calcium and phosphorus obtained from ingested food and exhibits anticancer effects, influencing various cancer types such as melanoma, colorectal cancer (CRC), and breast cancer (BC) [9].

In this sense, BC is the most prevalent cancer in women worldwide, constituting 79.7% of diagnosed cases in 2020, with a projected 17.9% increase by 2030 [10]. Geographic variations indicate higher incidences in Asia and Europe compared to Africa or Oceania [10], influenced by factors like alcohol consumption, lack of physical activity, and limited detection programs [11]. The complexity of BC involves interindividual variability and diverse tumor subtypes [12], prompting this review to explore intricate aspects through the current scientific literature. The pathogenesis, not fully understood, involves two main theories: stochastic and BC stem cell theory [13,14]. Ongoing research into mechanisms, particularly related to estrogen receptor-positive (ER+) and Human Epidermal Growth Factor Receptor 2-positive (HER2+) cancer, is crucial for effective prevention strategies [14,15]. While some risk factors like age, genetic predisposition, and breast density are non-modifiable, others, such as obesity and lifestyle, offer intervention opportunities in BC prevention [16,17,18]. Obesity, prevalent in contemporary society, contributes to higher estrogen levels and increased cancer cell growth [17]. Lifestyle choices, including alcohol consumption, also influence BC risk, affecting biological mechanisms [18]. Despite identified factors, uncertainties persist regarding the impact of geographic location, environmental pollution, and other variables on BC development [19,20].

The complex nature of the disease has led to the search for new prevention strategies, such as breast self-examination [21] or screening [22]. Additionally, studying the correlation between BC and vitamin D supplementation represents a promising avenue in the ongoing quest for effective prevention and early intervention strategies [23]. Therapeutic supplementation and fortification of food are commonly employed approaches to enhance vitamin D levels. Numerous intervention studies have shown the effectiveness of supplementing with vitamin D (either D2 or D3) through a single substantial dose or divided doses via oral and parenteral routes, leading to an increase in serum levels of the respective forms of 25(OH)D to different extents [24,25]. In BC, vitamin D plays a role in prevention and treatment by influencing cellular differentiation, inflammatory processes, and hormonal regulation [26]. Despite acknowledging this relationship’s importance, researchers disagree on the vitamin’s beneficial effects and optimal levels for disease prevention [27]. Various risk factors, both modifiable and non-modifiable, influence vitamin D’s effects on BC [28]. Interest in this relationship is growing, yet a consensus on its impact on disease prevention and development remains elusive. This study will explore the relationship between vitamin D supplementation and BC. In contrast to previous reviews in the field [29,30], the present analysis concentrates on recent articles, offering current perspectives to enhance our evolving comprehension of the subject.

Thus, this study aims to analyze in an integrative literature review the relationship between vitamin D levels and the prevention of BC in adult women within the current evidence framework, assessing the vitamin D levels that play a preventive role against BC, identifying the biological mechanisms of action, and the factors involved in this relationship.

## 2. Materials and Methods

### 2.1. Search Strategy

For this integrative literature review, a search strategy was designed to find the scientific literature on the relationship between vitamin D and BC. The publications were collected through an electronic search in the Web of Science, Scopus, and PubMed databases, using the MeSH terms “vitamin D”, “breast cancer”, “prevention”, “optimal levels”, “action mechanism”, and “risk factors” [31]. This was combined using the Boolean operator “AND”. The search strategy is represented in Table 1. The work was focused on figuring out the relationship between vitamin D levels in adult women and BC (1), the biological mechanism of action involved in the relationship between vitamin D and the prevention of BC appearance (2), and the potential risk factors that may influence the relationship between vitamin D and BC prevention (3). 

The inclusion criteria were as follows: articles addressing vitamin D supplementation in adult women aged 18 and older; articles providing detailed determinations of serum vitamin D levels; studies evaluating the effect of different risk factors involved in the relationship between vitamin D levels and breast cancer; and studies published within the last 5 years.

### 2.2. Statistical Analysis

The data extracted from the articles used as the working sample for the section “Relation of vitamin D level and breast cancer in adult women” [32,33,34,35,36] were the serum vitamin D levels considered significant in each of the articles. To carry out the analysis, the mean and standard deviation of the relevant data extracted from each study were calculated. The mean was calculated as the sum of the serum vitamin D concentration values divided by the number of values, providing an estimate of the representative value of the obtained results. Subsequently, the standard deviation was calculated using the square root of the mean of the differences.

## 3. Results and Discussion

**Figure 2:**
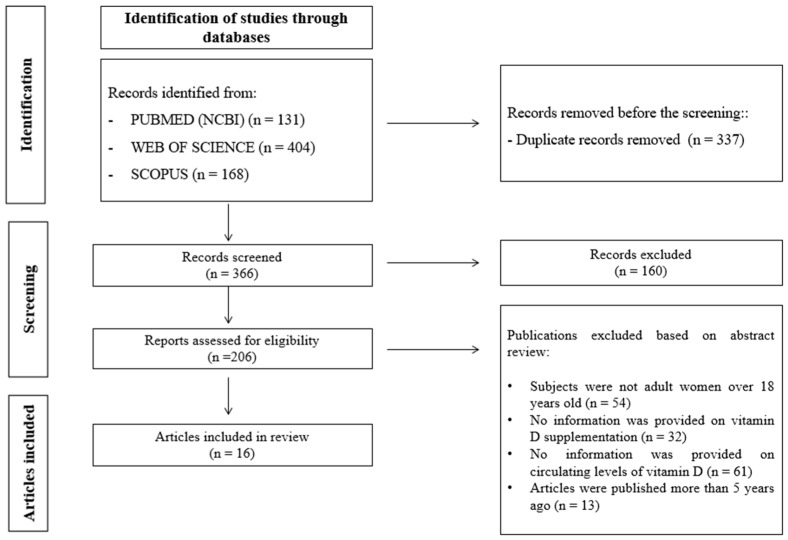
PRISMA flow diagram representing the study selection process [31].

For this integrative literature review, a series of articles of interest were selected for each objective set forth. Figure 2 illustrates the flowchart used for this work, detailing the steps and the study selection process. 

The initial step involved identifying articles by applying the specific keywords for each objective as outlined in Table 1, resulting in a total of 703 articles found in the scientific databases PubMed, Web of Science, and Scopus. Subsequently, duplicate articles were removed, leaving a total of 366 screened articles. In the third step, the selected records were evaluated for eligibility, resulting in the selection of 206 articles, while 160 publications were excluded as they were not deemed relevant for this work. Ultimately, a total of 16 studies for the three objectives were included in this work. 

### 3.1. Relationship between Vitamin D Levels and Breast Cancer in Adult Women

The relationship between vitamin D deficiency and BC is a subject of disagreement within the scientific community. Some researchers have highlighted the link between vitamin D deficiency and the subsequent development of BC [37], while other studies have failed to demonstrate such a relationship [27]. For this reason, this work first aims to analyze the link between vitamin D serum levels and the risk of developing BC through five studies [32,33,34,35,36], collectively evaluating the levels considered optimal for reducing the risk. These five studies comprise three case–control studies [32,35,36], a combined analysis of two randomized clinical trials and one prospective cohort [33], and a meta-analysis [34]. All these studies were conducted in adult women with the aim of analyzing serum levels of vitamin D and the risk of developing BC. The details of each study are summarized in Table 2 and discussed below. To evaluate the results, values deemed significant in protection against BC were selected, and simple statistical analyses were performed, calculating the mean and standard deviation of the chosen values from each study (Table 2). It was thus determined that the average serum concentration at which vitamin D could exert its protective effect against BC is 40.26 ng/mL with a deviation of 14.19 ng/mL (40.26 ng/mL ± 14.19 ng/mL).

Various authors have sought to highlight the importance of vitamin D in BC, as is the case with Voutsadakis et al. [38], who conducted a meta-analysis reporting on vitamin D levels in patients with newly diagnosed BC. They observed that the average serum level of vitamin D in BC patients was 26.88 ng/mL, while in control patients, it was 31.41 ng/mL; that is, vitamin D insufficiency could be related to the development or progression of BC. Another group of researchers, Shaukat et al. [39], support these findings, as they observed the relationship between vitamin D deficiency and the risk of developing BC in a population of women between 20 and 75 years old with serum levels of vitamin D below 20 ng/mL, similar to what was found by Voutsadakis et al. [38].

Fakour et al. [32], in their study, conducted a case–control trial where they examined a cohort of 140 women from an Iranian population, 70 cases and 70 controls, finding an inverse association between vitamin D and the risk of developing BC, with protective levels of 63.34 ng/mL. 

Although most studies reporting on the relationship between vitamin D and BC are conducted in relatively small cohorts, there are also large-scale studies where a reduction in the risk of developing BC is observed when there are sufficient levels of this vitamin [40].

One drawback encountered when conducting studies on this topic is the discrepancy in considering the optimal levels of vitamin D in BC prevention. Therefore, Lowe et al. [41] assessed the risk of developing BC using various concentrations of vitamin D, finding that women with the lowest levels had a risk five times greater than those with the highest levels. These findings are also observed in the article published by McDonnell et al. [33], where concentrations > 60 ng/mL in women compared to 20 ng/mL were associated with an 80% lower risk of developing BC. Furthermore, Crew et al. [42] utilized a case–control study conducted on 1026 cases and 1556 controls from a population of American women and found that serum levels of vitamin D ≥ 40 ng/mL were associated with a lower risk of BC.

Significant meta-analyses have also been conducted, such as the one carried out by Chen et al. [43], which includes an analysis of 21 studies on the intake and circulating levels of vitamin D and the risk of BC. The results obtained highlight a significant inverse relationship between vitamin D intake and the risk of BC, associated with a 45% lower risk. Hossain et al. [34] also conducted a meta-analysis in which they included a total of 22 studies, of which 14 specifically discuss serum vitamin D levels in relation to BC. They indicated that serum deficiency of this vitamin is associated with the onset of BC in the general population. Furthermore, they also highlight the importance of vitamin D supplementation or increased sunlight exposure for disease prevention.

To summarize, all these findings support that a serum vitamin D concentration ≥ 40.26 ng/mL (40.26 ng/mL ± 14.19 ng/mL) could be considered the level from which this vitamin exerts its protective effect against the development of BC. 

However, while previously discussed studies conclude the relationship between vitamin D deficiency and the risk of BC, there are also studies with other theories. Jacobs et al. [44] conducted a study aiming to associate circulating concentrations of vitamin D with the development of BC, studying a cohort of 3085 women, but found no evidence of a relationship between vitamin D concentrations and BC risk. Similarly, Visvanathan et al. [27] did not find this association in their study, which was conducted on 10 U.S. and 7 European cohorts.

The inconsistency of the results addressing vitamin D deficiency and BC is also evident in the study published by Edvardsen et al. [45], where they studied a total of 41,811 women, using adjustment for age, height, or body mass index (BMI), among other factors, and conducting a follow-up for 8.5 years. However, their results failed to establish a relationship between vitamin D deficiency and BC, as there were no significant differences between the values obtained. 

A meta-analysis published by Zhou et al. [46], which included eight trials with 72,275 patients, aiming to evaluate the value of vitamin D supplementation in BC prevention, concludes that this relationship is not significant, meaning that vitamin D does not reduce the risk of BC. This is contrary to the results obtained in the meta-analysis published by Chen et al. [43], where an association between the risk of BC and circulating levels of vitamin D could be observed.

As previously mentioned, BC is a very complex disease, partly due to the different tumor subtypes that exist. This is why various authors have studied not only the importance of vitamin D in BC risk but also the relationship of vitamin D deficiency with the most aggressive phenotypes of the disease.

An example of this is the work carried out by Lope et al. [35], who conducted a case–control study in a cohort of 1104 women, aiming to analyze serum vitamin D concentration and the risk of developing BC by pathological subtype. They found that lower serum levels of vitamin D could increase the risk of developing more aggressive phenotypes of BC. These results are also supported by Karthikayan et al. [36], who, conducting the same type of case–control study in a cohort of 156 women, also observed that lower levels of serum vitamin D correlate with more aggressive phenotypes of BC.

Similarly, Rainville et al. [47], in a clinical case study through the observation of 15 patients with triple-negative breast cancer (TNBC), the most aggressive form of BC, observed that the patients had lower average levels of vitamin D, suggesting that these are characteristic of this TNBC phenotype. However, information on this topic remains inconsistent within the scientific community. Such is the case with Rollison et al. [48], who conducted a case–control study. They observed that although there was evidence of the relationship between vitamin D and BC, they could not verify the connection between the deficiency of this vitamin and the risk of developing phenotypes like ER+/PR−.

As has been evidenced, there is no consensus on the serum levels of vitamin D that could be associated with a lower risk of developing BC. However, there are various studies, such as those mentioned earlier [33,38], that link a vitamin D concentration <20 ng/mL with a worse prognosis and progression of the disease. Furthermore, emphasizing the current scientific literature found in different databases, there are a higher number of studies that support the scientific evidence on the relationship between vitamin D deficiency and the risk of developing BC, as well as a higher predisposition to more aggressive phenotypes of the disease. This could represent a window for improving the prevention of this disease in women over 18 years old.

### 3.2. Biological Mechanism of Action Involved in the Relationship between Vitamin D and the Prevention of the Appearance of Breast Cancer

**Figure 3:**
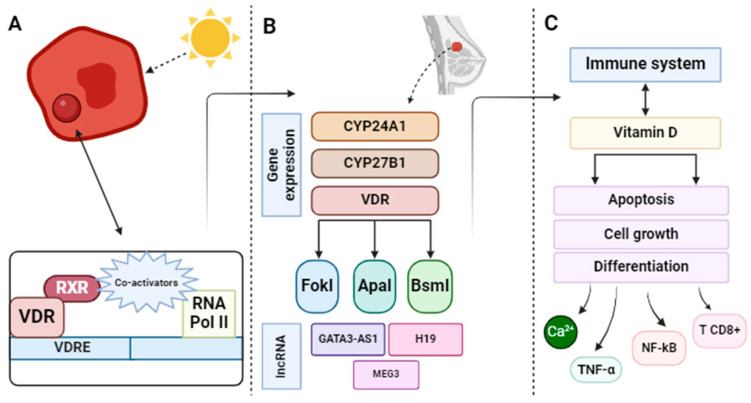
Representation of some of the different mechanisms of action involved in the relationship between vitamin D and breast cancer addressed in this study. (**A**) Representation of the vitamin D/VDR axis. (**B**) Representation of the regulation of genes related to breast cancer. (**C**) Representation of the mechanisms involved with the immune system. Abbreviations: VDR, vitamin D receptor; RXR, retinoid X receptor; VDRE, vitamin D response element; RNA Pol II, RNA Polymerase II; CYP24A1, Cytochrome P450 Family 24 Subfamily A Member 1; CYP27B1, Cytochrome P450 Family 27 Subfamily B Member 1; lncRNA, long non-coding RNA; MEG3, maternally expressed gene 3; Ca^+2^, calcium; TNF-α, tumor necrosis factor-alpha; NF-κB, nuclear factor kappa B; T CD8+, CD8 T lymphocytes.

BC is considered a truly complex disease, which is due, in part, to the existence of different tumor subtypes [12]. This leads to greater difficulty in elucidating the biological mechanisms underlying the relationship between vitamin D deficiency and the development of BC [49,50,51,52,53]. In this sense, the most relevant biological mechanisms that have been highlighted in the scientific literature involving vitamin D in BC are represented in Figure 3, summarized in Table 3, and described below, although only a small part of all those involved in the anticancer effect of this vitamin are known.

#### 3.2.1. Vitamin D/Vitamin D Receptor (VDR) Axis

In 1979, with the discovery of VDR in human mammary cancer cells, a new door was opened to study the relationship between vitamin D and BC carcinogenesis [54]. Currently, there is abundant scientific evidence supporting the relationship between vitamin D and the risk of BC. A key factor in this relationship is VDR, which is important because the active metabolite of vitamin D, 1,25(OH)2D, exerts its function by binding to it [55]. This receptor is present in virtually all tissues, including the lungs and the breast. Among the effects of VDR are the cessation of cell growth, differentiation, and apoptosis [56]. Thus, it has come under scrutiny as a regulator of growth modulated by vitamin D that could serve as a target for BC prevention.

VDR is characterized by its association with BC due to the presence of a series of polymorphisms, such as FokI (rs10735810), BsmI (rs1544410), ApaI (rs7975232), and TaqI (rs731236) [57]. This is because the VDR heterodimerizes with RXR, and the resulting complex binds to VDRE in the promoter region of target genes [58]. Thus, the polymorphisms can alter the main actions of VDR by affecting its binding to RXR and, consequently, to VDRE, indicating that the risk of BC in women may be influenced for this reason. 

Therefore, Raza et al. [49], in their study, observed that the polymorphisms specific to the VDR gene could cause alterations in the main actions of VDR, such as heterodimerization, thereby increasing the risk of developing BC. This would result in alterations in the biological function and circulating levels of vitamin D since the VDR receptor is essential for the effect of this vitamin. In their study, Santos-Martínez et al. [50] investigated the effect of 1,25(OH)2D, the active form of vitamin D, on ERα-BC. They observed that the complex formed by 1,25(OH)2D, VDR, and RXR binds to VDRE in the promoter region of the Erα gene, highlighting the importance of this complex and 1,25(OH)2D in the potential development of BC. This study provides insights into new preventive therapies for ERα-negative tumors by understanding their mechanism of action. Alimirah et al. [59] also studied this relationship, observing that PPARγ competes with VDR for binding to RXR in BC cells, thereby presenting another molecular mechanism that could be targeted in BC prevention. On the other hand, Friedrich et al. [60] also highlight the importance of the VDR-RXR-α complex in breast carcinomas, as they observed a higher expression of this complex in benign breast tissue. Thus, it could be suggested that this complex might serve as a chemopreventive agent for BC.

There are also opposing views to the results that identify VDR as essential in BC. Such is the case with Eliassen et al. [61], where they did not find significantly different associations in the expression of RXR for BC, nor a general association between plasma vitamin D and the risk of BC. They only found an inverse association between elevated levels of vitamin D and a lower disease risk in the summer. Here, plasma levels of the studied vitamin could indeed be related to a lower risk of tumors expressing high levels of VDR.

Despite the inconsistency of the results in the current scientific literature, it could be said that VDR plays a fundamental role in the biological mechanisms involved at the cellular level. Furthermore, more studies should be conducted on the relationship between VDR and the prevention of BC, as it appears to be a very interesting and, at the same time, largely unexplored field.

#### 3.2.2. Regulation of Genes Related to Breast Cancer

The effects produced by vitamin D in the body can be both non-genomic and genomic, meaning that genetics and genomics also play a significant role in the effects exerted by this vitamin [62]. As mentioned earlier, VDR is a member of the nuclear receptor superfamily, which is crucial for the biological actions of vitamin D, as it exerts its functions by binding to this receptor [63]. It is worth noting that there are numerous genes that are positively regulated (e.g., CYP24A1) or negatively regulated (e.g., CYP27B1) through the activation of VDR [64].

Current evidence links vitamin D deficiency and VDR polymorphisms with the risk of BC. For instance, Ahmed et al. [65] studied the prevalence of vitamin D deficiency and the implications of VDR genetic variations on BC risk in a case–control study involving 585 Ethiopian women. They found that the rs2228570 (FokI) polymorphism is associated with an increased risk of BC. These findings are further supported by the study conducted by Francis et al. [66], as they found that the rs2228570 polymorphism, as well as rs7041, were associated with a higher risk of BC. Moreover, they were able to elucidate that genetic factors could modify the tumor’s sensitivity to vitamin D. Another study conducted on an Iranian population of 311 women similarly concluded that the FokI polymorphism (rs2228570), as well as other polymorphisms such as BsmI (rs1544410) or ApaI (rs797532), in VDR were related to the risk of developing BC [67].

However, there are also studies that disagree about the effect of various VDR polymorphisms on vitamin D deficiency and the risk of BC. Such is the case with the study published by Dorjgochoo et al. [68], where they assessed the association between the risk of developing BC and 559 SNPs in 12 vitamin D-related genes, including 6 genes associated with the circulating level in a genome-wide association study (GWAS). The results suggest that VDR genetic polymorphisms do not play a significant role in the risk of BC in Chinese women. In another study, this time conducted on a U.S. population of 1329 women in a case–control study (845 controls and 484 cases), an inverse relationship was observed between VDR polymorphisms and the risk of BC in the BsmI (rs1544410) polymorphism of VDR, but not with FokI (rs2228570) [69]. This differs from the articles mentioned earlier [66,68]. In a meta-analysis published by Zhao et al. [70] and conducted by analyzing 25 studies on the relationship between VDR polymorphisms and BC, it was observed that, besides vitamin D levels being higher in controls than in cases in those studies, the FokI mutation is associated with a higher susceptibility to BC, while other mutations like BsmI or ApaI did not play a significant role. Thus, it can be concluded in this study that decreased vitamin D levels and the presence of FokI increase the likelihood of developing BC.

Other relevant genes involved in the relationship between vitamin D deficiency and BC are known as CYP24A1 and CYP27B1. The CYP24A1 gene is responsible for encoding the protein that initiates the degradation of the active form of vitamin D, 1,25-dihydroxyvitamin D3 [71]. Meanwhile, CYP27B1 encodes the enzymes that regulate the level of biologically active vitamin D and plays a significant role in calcium homeostasis [72]. A study published by Osanai et al. [73] underscores the significant role of CYP24A1 in BC. They found that vitamin D deficiency mediated by the intracellular metabolism of CYP24A1 leads to the development of malignant mammary neoplasms. Based on their results, these authors propose a strategy to silence CYP24A1 to reduce the risk of disease due to the gene’s impact on BC carcinogenesis. Another in vitro study [74] also supports this information. It observed a potential approach of inhibiting CYP24A1 to activate the vitamin D pathway in the prevention and treatment of BC. There are other studies that link BC with the CYP27B1 gene [75]. These studies observe that mammary cells express this cytochrome and that its action is inhibited with physiologically relevant concentrations of vitamin D. Thus, the link between vitamin D levels and the risk of developing the disease is observed when CYP27B1 is expressed. Latacz et al. [76] also studied the relationship of this gene with various pathologies, including BC. They found that the rs10877012 polymorphism is associated with vitamin D levels and the risk of BC. A study recently published by Dennis et al. [77] explores the relationship between vitamin D levels in BC cells and the expression of CYP24A1 and CYP27B1. Although they emphasize that these results are not yet well-established in the scientific literature, they did demonstrate that BC cells individually produce active vitamin D metabolites and highlight the expression of CYP24A1 and CYP27B1. Absolutely, understanding the intricate mechanisms of genes like CYP24A1 and CYP27B1 within mammary cells provides valuable insights. The correlation between low serum vitamin D levels and increased expression of these genes, subsequently elevating BC risk [78], emphasizes the significance of vitamin D in BC prevention. By delving deeper into these molecular pathways, it may be feasible to enhance existing prevention strategies, offering a more targeted and effective approach to reducing BC incidence and improving patient outcomes. Despite the increasing number of studies linking these genetic variants to vitamin D deficiency and the risk of BC, there is still an ongoing debate within the scientific community. The data obtained by different researchers remain inconsistent, emphasizing the need for further studies to support this information.

It is also important to mention the role of long non-coding RNAs (lncRNAs) in the pathogenesis of BC and vitamin D signaling. Blasiak et al. [51] highlight the role of these lncRNAs in BC, noting that a deeper study of GATA3-AS1, H19, or MEG3, among others, can elucidate the effects of vitamin D3 on BC mediated by lncRNA. However, these results have not yet been thoroughly demonstrated, as there are also many other lncRNAs that have not been identified. Nevertheless, it can be inferred that these lncRNAs play a significant role in the pathogenesis of BC [79].

In summary, there are various genes and genetic variants associated with circulating vitamin D levels and the risk of developing BC. While progress has been made in recent years, such as in the study of the role of VDR [66,68], there are still many inconsistencies and gaps in this research.

#### 3.2.3. Mechanisms Involved with the Immune System

Vitamin D is closely related to the immune response, as it stimulates the immune system to combat bacterial infections or prevent various autoimmune diseases [80]. Although they failed to demonstrate the fundamental role of vitamin D in BC, Negri et al. [81] were able to elucidate that this vitamin exerts anticancer effects through different mechanisms, such as the apoptosis of cancer cells and their growth. However, other authors [82] have not been able to determine the mechanisms by which vitamin D functions within the immune system in preventing BC, which may be due to the limited information available on the mechanisms related to BC risk and vitamin D, as well as the need for more specific studies. Another group of researchers, led by Gharib et al. [52], studied a population of 100 Saudi women aiming to associate serum vitamin D, calcium, interleukin-6 (IL-6), and tumor necrosis factor-alpha (TNF-α) with BC progression. They observed a significant association between the various factors and adverse prognostic characteristics of BC.

Staquicini et al. [83] published a study examining the role of vitamin D in the immune system in triple-negative breast cancer (TNBC) cells. What they found in this study was the presence of a peptide (CSSTRESAC) that binds to the vitamin D receptor, leading to a change in the profile of inflammatory cytokines, resulting in an antitumoral immune response mediated by vitamin D, reducing the risk of developing the TNBC phenotype. Other researchers focused on studying the relationship between vitamin D in the immune system and the development of ERα-negative BC [84]. In this case, it is observed that the combination of vitamin D and cytokines in ERα-BC elicits an immune response in the body. Therefore, these results could justify further studies in this field for the prevention of this tumor subtype. Despite the exhaustive search conducted in different scientific databases, no article discussing these observations on TNBC and ERα-BC in relation to vitamin D has been found. Therefore, further studies on this approach are considered important, as they could yield significant results in the prevention of such breast tumors.

Another factor that highlights the relevance of vitamin D in the immune system is its anti-inflammatory effect. Krishnan et al. [85] published a study where they reviewed different types of cancer, including BC, and the role of vitamin D or its active form, 1,25(OH)2D, in reducing inflammation. Thus, this study observed the inhibition of synthesis, the biological action of prostaglandins, and the suppression of nuclear factor kappa B (NF-κB) activation and signaling. Karkeni et al. [53] also found interesting results showing the importance of optimal levels of vitamin D in the body. They observed a higher number of CD8+ T lymphocytes in tumors treated with vitamin D, leading to increased recognition and destruction of malignant BC cells. This could potentially prevent the onset of the disease and, if it does develop, could also improve prognosis.

There are other mechanisms involved in the relationship between vitamin D and BC, such as the homeostasis of the redox balance in cells to prevent the development of malignant mammary neoplasms. This is highlighted in their publications by authors like Koren et al. [86], who reported on the effect of 1,25(OH)2D on the redox balance homeostasis in cells. This leads to the modulation of enzymes and transcription factors related to different phases of the cell cycle, such as apoptosis. However, there are also discrepancies about this biological mechanism, as some authors have obtained contrary results in their research where no significant associations were found between the action of vitamin D and the redox potential in BC [87]. 

It is important to understand the various mechanisms by which vitamin D interacts with the immune system, as it is known that deficient levels of vitamin D are associated with the manifestation of pathophysiological characteristics specific to BC [55]. However, the existence of various biological mechanisms involved in this context has been described that have not been thoroughly studied; hence, further research is needed.

### 3.3. Potential Risk Factors That May Influence the Relationship between Vitamin D and Breast Cancer Prevention

**Figure 4:**
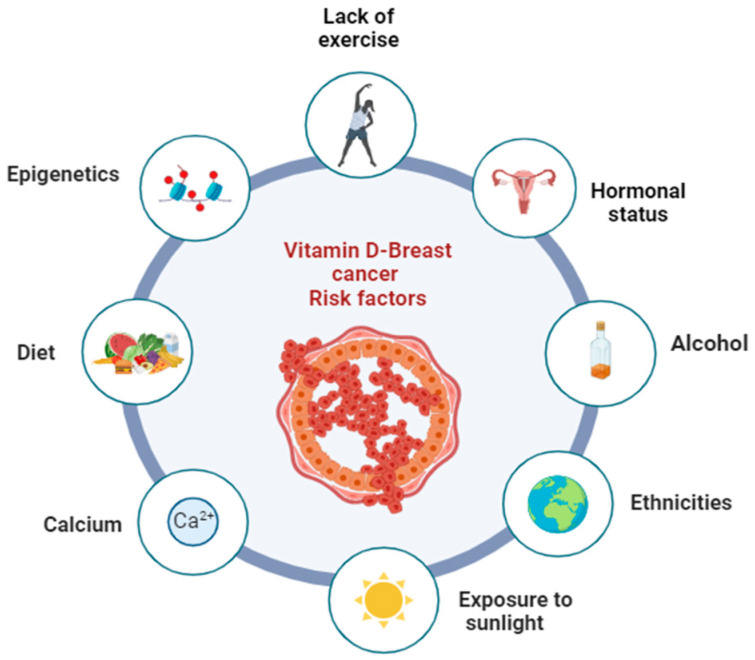
Representation of some of the factors influencing the relationship between vitamin D and breast cancer in the context of disease prevention mentioned in this study.

The relationship between vitamin D and BC in the context of prevention is a complex and multifactorial issue [28]. In this section, we will address some of the most relevant factors of this relationship (Figure 4).

Table 4 includes a set of five articles [88,89,90,91,92,93] aimed at elucidating various potential factors that may influence the relationship between vitamin D levels and BC. Various factors related to vitamin D and its influence on the risk of BC have been discussed, such as lifestyle [88], calcium [89], and epigenetics [90].

#### 3.3.1. Mechanisms Involved with the Immune System

Lifestyle, encompassing factors such as diet and exercise, plays a pivotal role in preventing various diseases, including BC. Diet is one aspect of lifestyle that can undergo significant changes, and it is well-established that a healthy diet is closely associated with a significant reduction in the risk of developing BC [94]. Buja et al. [88] explored the impact of consuming various foods and found that a high intake of meat or foods with a high glycemic index might be linked to an increased risk of BC. Conversely, a high consumption of vegetables or nutrients like calcium and vitamin D appeared to be inversely related to disease risk. Other researchers also emphasize the importance of increasing the intake of fruits, vegetables, and whole grains while reducing the consumption of meats, especially red meats, and saturated fats [95]. Romieu et al. [96] further highlight the significance of maintaining a healthy lifestyle, which includes a diet rich in vegetables, as well as legumes and fish. Moreover, a study by Rossi et al. [97] underscores the importance of consuming foods rich in polyphenols or phytoestrogens, products derived from soy, as well as fibers, which may have a protective effect against the onset of BC. 

However, while the evidence supporting the notion that a healthy diet reduces the risk of BC is becoming increasingly consistent, there are also authors who hold differing theories. For instance, Smith-Warner et al. [98] did not find significant associations between the consumption of carbohydrates and fats and an increased risk of BC. Similarly, findings by Michels et al. [99] explored dietary factors of particular interest in the context of BC, such as fat or vitamin intake, but did not identify a direct link between diet and BC, though they did find an association with alcohol consumption.

A healthy diet highly recommended by specialists is the Mediterranean diet, which is closely linked to BC prevention due to its significant number of fruits and vegetables and the consumption of healthy fats from olive oil [100]. For this reason, Montagnese et al. [101] conducted a study on women to observe the effects of the Mediterranean diet and exercise on BC survivors. They found that the combination of both factors, along with sufficient vitamin D levels, could enhance the quality of life for these women. Based on the discussion, it can be inferred that maintaining a healthy diet, such as the Mediterranean diet, helps maintain adequate serum levels of vitamin D, which in turn aids in BC prevention.

As previously mentioned, vitamin D deficiency is increasingly common today. Therefore, food fortification emerges as a preventive strategy to improve the nutritional status of the population and, consequently, could be established as a preventive measure in public health [7,102,103]. Cashman et al. [104] observed that fortifying various foods such as bread, milk, cheese, orange juice, or cereals can be used as a vehicle to enhance circulating concentrations of vitamin D in populations. On the other hand, Navarro Valverde et al. [105] also emphasize the importance of normalizing vitamin D levels through milk fortification. Santanatoglia et al. [106] developed innovative cheeses fortified with vitamin D3, such as giuncata and burrata, and found that this fortification could contribute to reducing the prevalence of vitamin D deficiency. Despite the findings in these articles, further studies are still needed to validate the relationship between vitamin D-fortified foods and BC, as mentioned earlier. It could be used as a vehicle to address this public health issue.

Exercise is also a crucial factor in maintaining a healthy lifestyle, which is related to the effects of vitamin D in BC prevention. Naderi et al. [107] conducted a study to observe how yoga training combined with high-dose vitamin D supplementation affected BC, finding an association between these factors. Other researchers, Campbell et al. [108], examined estrogens and androgens as biomarkers for postmenopausal BC risk. They observed that weight loss accompanied by moderate exercise reduced exposure to BC biomarkers. Similarly, Oliveira Sediyama et al. [109] conducted a study to evaluate the association between vitamin D levels and BC in women. They found that maintaining adequate levels of this vitamin, coupled with moderate physical activity, were considered protective factors against the disease.

Despite the studies, further research is still needed to correlate lifestyle factors, considering diet and exercise, with the risk of developing BC. What can be inferred is that maintaining healthy lifestyle habits is truly essential for the proper functioning of the body, as it can help prevent certain diseases and enhance people’s quality of life.

#### 3.3.2. Calcium

It is well established that the active form of vitamin D, 1,25(OH)2D, regulates calcium absorption, which plays a fundamental role in the metabolism of vitamin D [110]. This suggests that the interaction between vitamin D and calcium may be involved in the context of the risk of certain diseases, such as BC. Peila et al. [89] studied the effect of combined calcium and vitamin D (CaD) supplementation on the risk of ductal carcinoma in situ (DCIS) in the Women’s Health Initiative trial with a follow-up of approximately 20 years. They observed that CaD supplementation in postmenopausal women is associated with a reduced risk of DCIS, suggesting that continued use of this supplementation may have long-term health benefits. Similarly, Krusinska et al. [111] conducted a case–control study involving 420 women, aiming to analyze the serum concentration of vitamins and minerals and associate their relationship with BC prevention. They found that maintaining adequate serum levels of vitamin D and calcium, as well as iron, may have a protective effect against BC in postmenopausal women. Continuing this line of research, Lin et al. [112] evaluated calcium and vitamin D intake in relation to BC in premenopausal and postmenopausal women over a follow-up period of approximately 10 years. They observed that premenopausal women consuming higher amounts of calcium and vitamin D might have a lower risk of developing BC. These findings are also supported by results from Lipkin et al. [113], who found that low levels of calcium and vitamin D, combined with a high-fat diet, could have adverse effects on the mammary gland. Conversely, increasing calcium and vitamin D intake could have a chemopreventive effect against BC.

However, as is customary in the scientific community, there are also arguments against the relationship between vitamin D and calcium and BC. Such is the case with the results obtained by Qin et al. [114], who conducted a case–control study involving 1724 cases and 1233 controls to observe whether vitamin D and calcium levels correlated with a lower risk of ER-BC and TNBC in black women. They concluded that, ultimately, no association was established between CaD intake and the risk of ER-BC and TNBC subtypes, although they did find an association with vitamin D intake individually. Conversely, Fernández-Lázaro et al. [115] observed that calcium intake might be related to a lower risk of BC, except in premenopausal women, while for vitamin D intake, no association with a reduced risk of BC was found. Breast density is considered one of the most important risk factors for BC [16]. Thus, Bertone-Johnson et al. [116] studied how CaD supplementation for one year could affect the breast density of 330 postmenopausal women. However, what they found was that the effect of CaD supplementation on reducing breast density could not be demonstrated, and therefore, it is not related to a lower risk of BC, but they emphasize that further studies are needed to explain this potential interaction.

Studies addressing CaD supplementation in the context of BC prevention are indeed scarce. Most articles in the scientific literature focus on the study of supplementation of both factors separately [117,118], so combined CaD supplementation and its association with BC risk require further research.

#### 3.3.3. Other Factors

As previously mentioned, the relationship between vitamin D and BC in the context of prevention is a complex and multifactorial issue [28]. Factors such as supplementation, calcium, or lifestyle are some that we have already discussed, but there are still many others.

Epigenetics can be defined as the set of genome modifications that are heritable, reversible, and do not alter the nucleotide sequence. The main epigenetic modifications include DNA methylation, histone modifications, and the regulation and expression of microRNAs (miRNAs). It is considered an important factor because it results in the modification of gene expression and, consequently, of proteins, so they are involved in different phases of the cell cycle, such as growth or differentiation [119]. Various authors have highlighted the importance of methylation in the relationship between vitamin D and BC, as the methylation levels of some genes, such as RXR, or hypermethylation of the VDR promoter, could affect vitamin D levels and the risk of developing BC [90,120,121,122]. Furthermore, epigenetics can also be related to factors such as diet [123]. Other epigenetic modifications may also be related to vitamin D levels and the risk of developing BC, such as histone methylation [50,124] and the regulation and expression of miRNAs [125,126,127].

Another factor that is increasingly strongly associated with vitamin D levels and the reduction of BC risk is exposure to sunlight. This is important since vitamin D predominantly comes from UVB radiation, which provides between 50% and 90%, while the rest comes from the diet [128]. Increased exposure time to sunlight may reduce the risk of BC, and this could be explained by increased vitamin D production in the body [91,129,130]. This factor, in turn, can be influenced by factors such as latitude [131,132,133] or the season of the year [134].

Furthermore, hormonal status is also a significant factor in this relationship, as women undergo various modifications in their hormone levels throughout their lives, and it is known that vitamin D plays a fundamental role in this hormonal state [135,136]. Several researchers have found an inverse association between blood vitamin D levels and the risk of developing BC, both in premenopausal and postmenopausal women [92,137,138,139].

To summarize, existing differences among races and ethnicities worldwide are also a significant factor. An example of this is that it has been observed that black women have a higher susceptibility to BC, specifically the more aggressive phenotypes. This may be due to different genetic backgrounds, such as dark skin pigmentation, which results in a high concentration of melanin that inhibits the synthesis of vitamin D, acting as a barrier to UVB rays from the sun [140,141]. This leads to lower serum levels of vitamin D in the bodies of these women, increasing the risk of developing BC [93,142,143].

## 4. Conclusions

From the results analyzed in this study, the deficiency of vitamin D is closely associated with the development of BC. In general, higher serum levels of vitamin D may exert a protective effect against the development of the disease. In the present study, it has been observed and discussed that serum levels of vitamin D ≥ 40.6 ng/mL ± 14.19 ng/mL could be considered protective against the risk of developing BC.

In addition, there are different biological mechanisms involved in the relationship between this pathology and vitamin D. This includes those related to VDR, where polymorphisms, especially FokI (rs2228570) and the complex it forms with RXR (VDR-RXR), are closely linked to BC. Additionally, genes such as CYP24A1 and CYP27B1 are implicated in this relationship, as well as lncRNAs GATA3-AS1, H19, or MEG3. Finally, it is noteworthy that immune system mechanisms are involved, as vitamin D may exert anticancer effects through cell cycle-related mechanisms, and factors such as IL-6 or TNF-α reduce inflammation in the tumor microenvironment, maintaining redox balance and preventing the development of malignant breast neoplasms.

Scientific evidence also exists regarding various risk factors that can affect the relationship between BC and vitamin D deficiency. For instance, diet plays a role, and maintaining a healthy diet like the Mediterranean diet is inversely related to BC development. Therefore, the fortification of foods with vitamin D, such as bread, milk, cheese, or cereals, has gained prominence as a prevention strategy against vitamin D deficiency. Additionally, engaging in moderate exercise contributes to this relationship. Another key factor is calcium, as joint supplementation with vitamin D (CaD) has been associated with a lower risk of BC. Finally, the importance of epigenetics, exposure to sunlight, hormonal status, and racial and ethnic differences should be mentioned.

Despite the data presented and discussed in this work, further studies are still needed to highlight the importance of vitamin D deficiency in BC risk, as well as the mechanisms and factors involved in this relationship. Understanding this information could lead to significant advances in BC prevention strategies, resulting in a substantial public health benefit.

## Figures and Tables

**Table 1:** Search strategy used in the different databases.

Objective	Search Strategy	Total Results
1	“Vitamin D” AND “Breast cancer” AND “Prevention”	*n* = 467
	“Vitamin D” AND “Breast cancer” AND “Prevention” AND “Optimal levels”	*n* = 14
2	Vitamin D” AND “Breast cancer” AND “Prevention” AND “Action mechanism”	*n* = 30
3	“Vitamin D” AND “Breast cancer” AND “Prevention” AND “Risk factors”	*n* = 192
	*N* = 703	

**Table 2:** Serum vitamin D levels that may influence breast cancer risk.

Sample Size	Design	Significant Serum Vitamin D Levels (ng/mL)	Results	References
144 (71 cases/73 controls)	Case–control study	63.34	The average level of vitamin D is 39.04 ng/mL and 63.34 ng/mL in cases and controls, respectively (*p* = 0.046). An inverse and independent association between vitamin D and BC is observed.	[32]
5038 (77 cases/4961 cases)	Combined analysis of two randomized clinical trials and one prospective cohort	42.5	80% less risk of BC at concentrations >60 ng/mL compared to <20 ng/ml.	[33]
123,044 (25,515 cases/97,529 controls)	Meta-analysis	27.07	Direct association between vitamin D deficiency and BC (RR = 1.91; 95% CI = 1.5 to 2.41; *p* < 0.001).	[34]
1104 (546 cases/558 controls)	Multicase–control study	31	Vitamin D levels above 27 ng/mL were associated with a 12% lower risk of postmenopausal BC. Significant dose–response trends (OR per 10 nmol/L = 0.88, 95% CI = 0.82–0.94). More pronounced protection in triple-negative tumors.	[35]
156 (78 cases/78 controls)	Case–control study	37.41	Mean serum levels of vitamin D in cases are significantly lower compared to controls (22.33 ± 8.19 vs. 37.41 ± 12.9 ng/mL, respectively; *p* = 0.0001).	[36]
Average			40.26 ± 14.19 ng/ml	

ng/mL, nanograms per milliliter; BC, breast cancer; RR, relative risk; CI, confidence interval; OR, odds ratio.

**Table 3:** Mechanisms of action involved in the relationship between vitamin D and breast cancer prevention.

Biological Mechanism	Objective	Conclusion	References
Vitamin D/VDR axis	To study the distribution of the FokI polymorphism (rs2228570) in VDR and its association with BC	The risk and pathogenesis of BC can be influenced by various VDR SNPs, including FokI	[49]
Vitamin D/VDR axis	To investigate the mechanisms involved in the expression of ERα-negative	The induction of ERα dependent on 1,25(OH)2D in ERα-negative BC cells results from the binding of the VDR-RXR complex to VDRE in the promoter region of the ERα gene, including negative regulation of enzymes with chromatin remodeling activities	[50]
Regulation of genes related to BC	Providing arguments that vitamin D/VD3 may include protective effects on BC through the modulation of lncRNA that are important for BC pathogenesis	The involvement of different lncRNAs in the interplay between vitamin D3/VDR signaling and the pathogenesis of BC has been identified, such as GATA3-AS1, H19, or MEG3	[51]
Mechanisms involved with the immune system	Studying the association between serum vitamin D, calcium, IL-6, TNF-α, and chemerin and the progression of BC	Higher levels of IL-6, TNF-α, and chemerin were significantly associated with the presence of BC, particularly in its more advanced stages	[52]
Mechanisms involved with the immune system	Evaluating the impact of vitamin D on the progression of BC and the microenvironment of the mammary tumor	Vitamin D can modulate the growth and inflammation of BC tumors in the in vivo tumor microenvironment, as an increase in CD8+ T cells was observed	[53]

VDR, vitamin D receptor; BC, breast cancer; ERα, estrogen receptor-alpha; RXR, retinoid X receptor; VDRE, vitamin D response element; lncRNA, long non-coding RNA; IL-6, interleukin-6; TNF-α, tumor necrosis factor-alpha.

**Table 4:** Description of studies showing factors influencing the relationship between vitamin D and breast cancer prevention.

Risk Factor	Objective	Conclusion	References
Lifestyle	Study on health policies and healthy dietary practices for the prevention of BC	A healthy lifestyle may be associated with a significant reduction in the risk of BC	[88]
Calcium	Studying the effect of CaD supplementation on the risk of DCIS	Supplementation with CaD in postmenopausal women is associated with a reduced risk of DCIS	[89]
Epigenetics	Studying the relationship between vitamin D, DNA methylation, and BC	The methylation of CpG sites in genes related to vitamin D may interact with 25(OH)D to influence the risk of BC	[90]
Exposure to sunlight	Examining the relationship between exposure to sunlight and a lower risk of BC	Greater exposure to solar radiation is associated with a decreased risk of BC	[91]
Hormonal status	Assessing the association between vitamin D levels and BC in premenopausal and postmenopausal women	BC risk is inversely related to vitamin D levels, with no differences between premenopausal and postmenopausal women	[92]
Ethnicities	Studying the association between vitamin D and BC in African American/Black and Hispanic/Latina women	Vitamin D may have a protective effect against BC, including in African American/Black and Hispanic/Latina women	[93]

BC, breast cancer; CaD, calcium and vitamin D supplementation; DCIS, ductal carcinoma in situ; DNA, deoxyribonucleic acid.

## Data Availability

Data sharing is not applicable to this article as no new data were created or analyzed in this study.
